# Antibacterial and Antibiofilm Activity of *Clerodendron Cyrtophyllum* Turcz. Ethanolic Extracts against *Staphylococcus aureus* from Bovine Mastitis

**DOI:** 10.4014/jmb.2506.06007

**Published:** 2025-11-27

**Authors:** Guoshun Pei, Rong Cao, Qiguo Li, Yue Hu, Yakun Zhang, Yao Feng, Yanrong Zeng, Yudie Guo, Yehui Luo, Lina Liu, Chengjian Tan

**Affiliations:** 1 Shool of Ethnic Medicine, Guizhou Minzu University, Guiyang 550025, P.R. China; 2 Engineering Research Center of Cellular Immunotherapy of Guizhou Province, Key Laboratory of Infectious Immune and Antibody Engineering of Guizhou Province, School of Biology and Engineering (School of Health Medicine Modern Industry)/School of Basic Medical Science, Guizhou Medical University, Guiyang 561113, P.R. China; 3 Key Laboratory of Environmental Pollution Monitoring and Disease Control, Ministry of Education, Guizhou Medical University, Guiyang 561113, P.R. China; 4 Key Laboratory for the Development and Utilization of Guizhou Minority Medical Resources (Guizhou Minzu University), State Ethnic Affairs Commission, Guiyang 550025, P.R. China

**Keywords:** *Clerodendron cyrtophyllum* Turcz., *Staphylococcus aureus*, bovine mastitis, antibacterial activity, membrane permeability, antibiofilm activity

## Abstract

Bovine mastitis causes huge economic losses for the dairy industry worldwide. *Staphylococcus aureus* (*S. aureus*) is an important pathogen that induces bovine mastitis, and its resistance to antibiotics has become a severe problem in bovine mastitis therapy. Therefore, the development of new treatments for this potentially fatal infection is urgently needed. *Clerodendron cyrtophyllum* Turcz. is a traditional Chinese medicine that has been used for the treatment of various diseases. However, its antibacterial effect on *S. aureus* in bovine mastitis is rarely investigated. In this study, *Clerodendron cyrtophyllum* ethanolic extract (CTE) was prepared, and its *S. aureus*-inhibting activities and antibiofilm effects were determined. The mechanisms of CTE against *S. aureus* were also investigated. The results showed that the growth of *S. aureus* was inhibited by CTE, and the minimum inhibitory concentration (MIC) of CTE was 250 μg/ml. After treatment with CTE, extracellular alkaline phosphatase (AKP), protein, and nucleic acid of *S. aureus* were increased, suggesting that the permeability of *S. aureus* cells was enhanced. Furthermore, intracellular reactive oxygen species (ROS) in *S. aureus* were increased, suggesting that CTE inhibited *S. aureus* growth by causing oxidative damage. In addition, CTE treatment suppresses biofilm formation of *S. aureus*, as almost 50% of biofilm was scavenged at 2× (MIC) of CTE. After CTE treatment, *icaA*, *sarA*, and *sigB* mRNA levels were significantly downregulated, whereas the *icaR* mRNA level was significantly upregulated, indicating that CTE suppressed biofilm formation by regulating expression of the biofilm formation-related genes. The findings of this study indicate that CTE could be a potential treatment for bovine mastitis-associated *S. aureus*.

## Introduction

Bovine mastitis is a common mammary gland disease of dairy cows and causes great economic losses in the dairy industry worldwide [1, 2]. *Staphylococcus aureus* (*S. aureus*) is one of the major infectious bacteria that induce bovine mastitis [3-5], while antibiotics are currently the dominant treatment strategy for this disease [6]. However, due to the overuse of antibiotics, pathogens such as *S. aureus* have increasingly developed resistance to them [7]. A previous study reported that almost 90% of *S. aureus* strains have developed antibiotic resistance, resulting in the reduced effectiveness of these drugs [8].

Approximately less than 0.1% of all microbial populations are in planktonic growth mode, with most bacteria being organized into more complex structures known as biofilms [9]. Biofilm formation reduces by 1,000-fold the susceptibility of bacteria to most antibiotics [10]. Forming biofilms is one of the main methods used by *S. aureus* to keep causing infections [11]. Due to poor therapeutic efficacy in bovine *S. aureus* mastitis, the development of new antibacterial medicines that may inhibit both planktonic cell growth and biofilm formation has become increasingly urgent. Herbal antimicrobials have a long history of use in Chinese medicine, and they are applied in the treatment of many types of infections [12]. *Clerodendron cyrtophyllum* Turcz. is a plant belonging to the Verbenaceae family [13]. In China, the plant is known as “da qing,” where it is mainly used to treat inflammation and bacterial infections [14, 15]. At present, both phenolic acids and flavonoids with antimicrobial activity have been isolated from *C. cyrtophyllum* [16-18].

Investigations on *C. cyrtophyllum* suggest that it has potential as an antibacterial agent, although further research is required. Therefore, our goal in the present study was to investigate the antibacterial activity and antibiofilm effect of *C. cyrtophyllum* Turcz. ethanol extract (CTE) against *S. aureus* from bovine mastitis, and to conduct a preliminary evaluation of its effects on *S. aureus* mastitis in a mouse model, to provide scientific evidence for the use of CTE in treating bovine *S. aureus* mastitis.

## Materials and Methods

### Strain Identification

*S. aureus* used in this study was isolated from bovine with clinical mastitis and characterized by molecular biology approaches (Figs. S1 and S2).

### Preparation of *Clerodendron cyrtophyllum* Turcz. Ethanol Extract (CTE)

*Clerodendrum cyrtophyllum* Turcz. was collected in Guiding County, Guizhou Province, China in June 2021, and was identified by Kunming Biotechnology Co., Ltd. (China). The voucher specimen (GZMU2021D02) was deposited in the Natural Products Chemistry Laboratory, Guizhou Minzu University, China. The dried, whole plant (100 g) was extracted with 500 ml 95% EtOH (× 3) under reflux conditions for 3 h to obtain 10.98 g of CTE.

### Measurement of the Inhibitory Zone Diameter

As a medium, 15 ml of Mueller Hinton broth (MHB) containing 1.5% agar was cooled to 50°C, and then poured into a plate. When the agar had cooled and solidified, 100 μl of *S. aureus* suspension was added to the plate and the agar surface was swabbed with a sterile stainless steel spreader. Sterile filter papers with a diameter of 6 mm were infused with 10 μl of CTE solution (400, 200, 100, 50, 25 μg/ml), 99% dimethyl sulfoxide (DMSO), and erythromycin (50 μg/ml), respectively. The filter papers were then placed on the agar plate and incubated at 37°C for 24 h, the diameters of inhibitory zones were measured, and photographs were taken.

### Minimal Inhibitory Concentration (MIC) Test

The MIC of CTE was measured by the broth microdilution method. The suspension density of the overnight *S. aureus* culture was adjusted to 0.5 McFarland standard. The *S. aureus* solution was then diluted (1:30) with MHB medium, and 0.1 ml of the diluted *S. aureus* solution (~5×10^7^ colony forming units (CFU)/ml) was added to a 96-well plate with gradient descending concentrations of CTE. After incubation at 37°C for 24 h, the results were observed, and the MIC was defined as the lowest concentration of CTE yielding no visible growth.

### Growth Curve Analyses

Overnight *S. aureus* culture was adjusted to 5 × 10^6^ CFU/ml in fresh MHB medium, and 100 μl of the diluted *S. aureus* solution was added to the 96-well plate. After CTE was added to reach the concentrations of 1/2 × MIC, 1 × MIC, and 2 × MIC, the plate was incubated for 24 h at 37°C. The optical density at 595 nm (OD_595_) was read at 0, 4, 8, 12 and 24 h. Then, a growth curve was drawn with OD_595_ as the vertical coordinate, and culture time as the horizontal coordinate.

### Biofilm Analysis

The biofilm formation of *S. aureus* was analyzed by crystal violet staining. The steps for biofilm inhibition were as follows: overnight *S. aureus* culture was diluted to 5 × 10^6^ CFU/ml with fresh MHB medium, and then added into the 96-well plate (100 μl/well) containing various concentrations of CTE (1/2 × MIC, 1 × MIC, and 2 × MIC). *S. aureus* solution without CTE was set as the negative control. After the 96-well plate was incubated at 37°C for 24 h, the supernatant was discarded, and the wells were washed 3 times with sterile phosphate-buffered saline (PBS) to remove planktonic *S. aureus* cells. After the plate was dried at room temperature, the adherent biofilms were fixed with 4% paraformaldehyde (200 μl), and then stained for 20 min with 0.1% crystal violet (200 μl). Finally, each well was added with 200 μl of 30% glacial acetic acid. The optical density at 492 nm (OD_492_) was measured using a Cytation 5 Cell Imaging Multimode Reader (BioTek, USA) to test the biomass of biofilms and capture images. For biofilm disruption, *S. aureus* was cultured overnight, and the concentration was adjusted to 5×10^6^ CFU/ml before being added into the 96-well plates (200 μl/well) and incubated at 37°C for 24 h to allow for mature biofilm formation. The mature biofilms were treated with different concentrations of CTE (1/2 × MIC, 1 × MIC, and 2 × MIC), and subsequently incubated at 37°C for another 24 h. After that, each well was rinsed once with PBS (50 μl/well), and the reduction of biofilm biomass was measured using the Cytation 5 Cell Imaging Multimode Reader.

### Detection of Viable *S. aureus* in Biofilms

The overnight cultured *S. aureus* was diluted to 5 × 10^6^ CFU/ml and placed into the 96-well plates (100 μl/well supplied with 1/2 × MIC, 1 × MIC, and 2 × MIC of CTE), followed by cultivation at 37°C for 24 h. After discarding the medium and washing with 0.9% sterile sodium chloride 3 times, each well was added with 190 μl of 0.9% sterile sodium chloride and 10 μl of CCK8, and then cultured for 2 h at 37°C. The Cytation 5 Cell Imaging Multimode Reader was used to record the optical density at 450 nm (OD_450_).

### Membrane Permeability Assays

The membrane permeability was determined by protein and nucleic acid leakage. *S. aureus* in the log phase was collected by centrifugation (6,500 ×*g* , 10 min), and the bacterial precipitate was washed thrice with PBS. Subsequently, *S. aureus* suspensions were treated with different concentrations of CTE (1/2 × MIC, 1 × MIC, and 2 × MIC), the drug solvent (0.1% DMSO) was used as control, and the mixtures were cultured in an incubator shaker at 37°C and 160 r/min for 5 h. After that, the cultures were taken at the time points 6.0, 8.0 and 10.0 h, and centrifuged (6,500 ×*g*, 10 min). The cultured supernatants were then collected and the absorbances were measured at wavelengths of 260 nm or 280 nm by ultraviolet and visible spectrophotometry.

### Determination of CTE on Alkaline Phosphatase (AKP) Activity of *S. aureus*

The extracellular AKP activity was used to evaluate the effect of CTE on the *S. aureus* cell wall. *S. aureus* in the log phase were collected by centrifugation (6,500 ×*g*, 10 min), and the precipitate was washed 3 times with PBS. Subsequently, the suspensions of *S. aureus* cells were treated with various concentrations of CTE (1/2 × MIC, 1 × MIC, and 2 × MIC), and then cultured in an incubator shaker at 37°C, 160 r/min for 1 h. After that, the cultures (5 ml) were taken at different time points and centrifuged (6,500 ×*g*, 10 min). The culture supernatants were collected and added with 0.3 ml p-nitrophenyl phosphate (pNPP, 1 g/L), followed by incubation in the dark at 20°C for 20 min. Subsequently, the addition of 0.2 ml sodium hydroxide (3 M/L) stopped the reaction. The optical densities at 405 nm (OD_405_) were then determined by ultraviolet and visible spectrophotometry.

### Determination of Reactive Oxygen Species (ROS)

Intracellular ROS was probed using 2,7-dichlorodihydrofluorescein diacetate (DCFH-DA). Briefly, the log phase *S. aureus* cells were mixed with DCFH-DA (10 μmol/L), incubated for 20 min, and then placed into the 96-well plates (100 ml/well). After treatment with or without CTE (1/2 × MIC, 1 × MIC, and 2 × MIC) for 1 h, the levels of ROS in *S. aureus* cells were tested every 0.5 h at fluorescence excitation/emission wavelengths of 488 nm/ 525 nm by using a BioTek Synergy H1 microplate reader (USA).

### RNA Extract, Complementary DNA (cDNA) Synthesis, and Reverse Transcription Quantitative Polymerase Chain Reaction (RT-qPCR)

*S. aureus* at log phase treated with CTE (1 × MIC) for 24 h was collected for analysis of gene expression by RT-qPCR. Total RNA was extracted from *S. aureus* by using the TRIzol reagent (Invitrogen, USA). TB Green Premix Ex Taq II (Tli RNaseH Plus), ROX Plus (Takara, China) was used to perform the RT-qPCR following the manufacturer's instructions. The thermocycler conditions were as follows: 95°C for 30 sec, then 40 cycles at 95°C for 10 sec, 55°C for 30 sec, and 72°C for 30 sec. Primers were used in this study as previous reported [19], with *rpoB* as internal control (Table 1).

### Statistical Analysis

Software Graphpad Prism 9.0 was used for statistical analysis. Statistical differences were conducted using *t*-tests. Data are expressed as the mean values ± SD. Values of *p* <0.05 were considered to indicate statistical significance.

## Results

### Inhibitory Zone Diameter of CTE

**Fig. 1:**
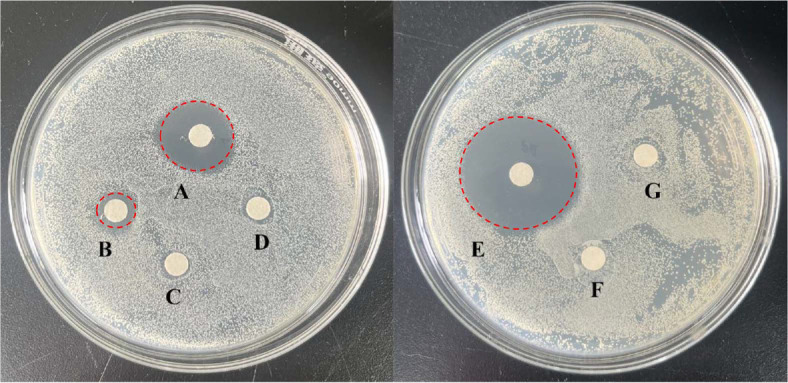
Inhibitory zone of CTE against *S. aureus*. (**A**) CTE (400 μg/ml); (**B**) CTE (200 μg/ml); (**C**) CTE (100 μg/ml); (**D**) CTE (50 μg/ml); (**E**) Erythromycin (50 μg/ml); (**F**) CTE (25 μg/ml); (**G**) DMSO. *n* = 3 in each group.

The antibacterial effect of CTE on *S. aureus* was tested and compared with erythromycin (as positive control) and DMSO (as negative control). As shown in Fig. 1, the antibacterial activity of CTE against *S. aureus* was compared with DMSO as follows: at 400 μg/ml, CTE showed moderate inhibitory effect (the average inhibitory zone diameter was 13 ± 0.35 mm); at 200 μg/ml, CTE showed low inhibitory effect (the average inhibitory zone diameter was 7 ± 0.32 mm); when the concentration was lower than 200 μg/ml, CTE had no obviously inhibitory effect on *S. aureus*, whereas erythromycin (50 μg/ml) exhibited high antibacterial activity (the average inhibitory zone diameter was 24 ± 0.47 mm) (Table 2). The results suggested that CTE (400 μg/ml) obviously inhibited the growth of *S. aureus*, although the inhibitory effect is less than that of erythromycin (50 μg/ml).

**Note:** An inhibitory zone diameter less than 7 mm indicated no inhibitory effect; a diameter between 7 and 10 mm indicated low sensitivity; a diameter greater than 10 mm but less than 20 mm indicated moderate sensitivity; a diameter greater than 20 mm indicated high sensitivity [20].

### MIC of the CTE

**Fig. 2:**
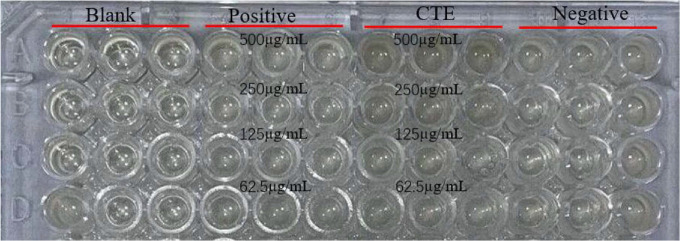
MIC of CTE against *S. aureus*. Note: Blank: 200 μl MHB; Positive group: 100 μl *S. aureus* suspension + 100 μl different concentrations of Ampicillin; CTE group: 100 μl *S. aureus* suspension + 100 μl different concentrations of CTE; Negative group: 100 μl *S. aureus* suspension + 100 μl MHB; *n* = 3 in each group.

The MIC of CTE against *S. aureus* was tested by broth microdilution methods. CTE and ampicillin (positive control) were diluted to 500, 250, 125, and 62.5 μg/ml, respectively, and then cultured with *S. aureus* at 37°C for 24 h. MHB was used as a blank control, while MHB plus bacterial suspension acted as a negative control. The inhibition rate was estimated by comparing turbidity in the presence or absence of CTE. The growth of *S. aureus* was inhibited by CTE at an MIC of 250 μg/ml, whereas the MIC of ampicillin to *S. aureus* was less than 62.5 μg/ml (Fig. 2).

### Effect of Different Concentrations of CTE on the Growth Curves of *S. aureus*

**Fig. 3:**
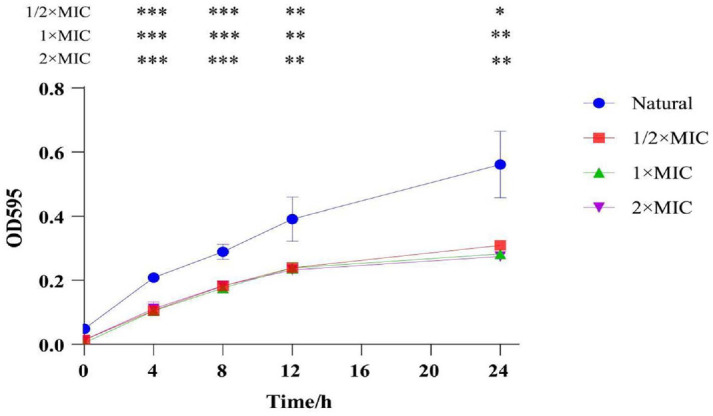
Growth curve of *S. aureus* after CTE treatment. The planktonic growth of *S. aureus* was monitored under different concentrations of CTE, including 0 × MIC (natural), 1/2 × MIC, 1 × MIC, and 2 × MIC. Data are expressed as the mean values ± SD, and *n* = 3 in each group;*p < 0.05, **p<0.01, ***p < 0.001, vs. the natural.

The inhibitory activity of CTE on the growth of *S. aureus* was evaluated. As shown in Fig. 3, the growth of *S. aureus* was significantly inhibited by CTE at concentrations of 1/2 × MIC, 1 × MIC, and 2 × MIC. The results suggested that CTE is a potential antibacterial agent against *S. aureus*.

### Antibiofilm Effect of CTE

**Fig. 4:**
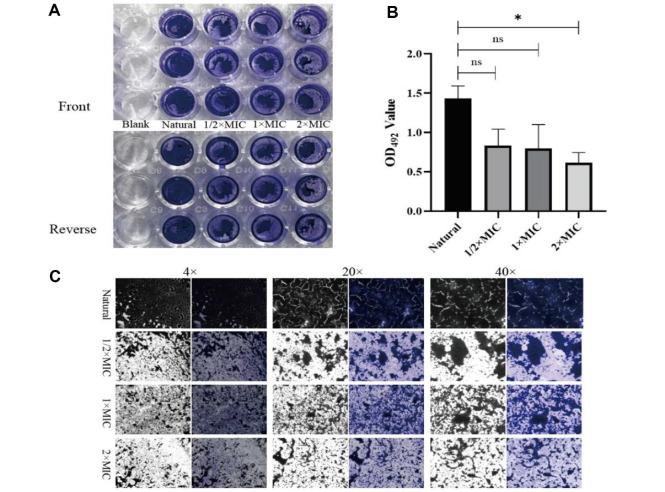
CTE inhibition of *S. aureus* biofilm formation. (**A**) Crystal violet staining; (**B**) Biofilms were dissolved by glacial acetic acid and OD_492_ values were measured. Data are expressed as the mean values ± SD, and *n* = 3 in each group; *p < 0.05, ns: no statistical difference, vs the natural. (**C**) Images at different magnifications were captured by Cytation 5 Cell Imaging Multimode Reader. The left-hand side is a black/white bright field, and the right-hand side is a color field.

Crystal violet staining was performed for quantitative analysis of the antibiofilm efficacy of CTE. The results suggested that CTE inhibited the biofilm formation of the tested *S. aureus*, and resulted in a significant decrease at the concentration of 2 × MIC, with almost 50% of biofilm being scavenged (Fig. 4A and 4B). A visible biofilm inhibition by 2 × MIC of CTE was also observed in the Cytation 5 images (Fig. 4C), consistent with the crystal violet staining results.

**Fig. 5:**
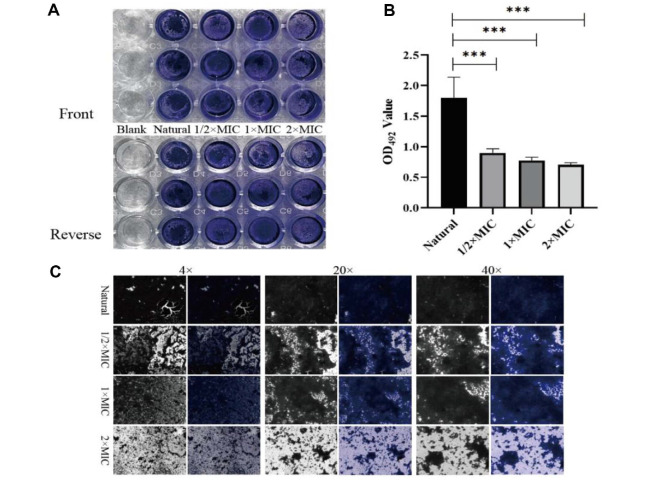
CTE destruction of mature biofilms of *S. aureus*. (**A**) Crystal violet staining; (**B**) Biofilms were dissolved by glacial acetic acid and OD_492_ values were measured. Data are expressed as the mean values ± SD, and *n* = 3 in each group; *p < 0.05, ns: no statistical difference, vs. the natural. (**C**) Images at different magnifications were captured by Cytation 5 Cell Imaging Multimode Reader. The left-hand side is a black/white bright field, and the right-hand side is a color field.

The effect of CTE on mature biofilm was also measured by crystal violet staining. The results indicated that concentrations of 1/2 × MIC, 1 × MIC, and 2 × MIC of CTE led to significant decrease of the mature biofilms (Fig. 5A and 5B). Visible mature biofilm reductions caused by 1/2 × MIC, 1 × MIC, and 2 × MIC of CTE were observed in the Cytation 5 images (Fig. 5C), consistent with the crystal violet staining results.

### CTE Inhibits *S. aureus* Embedded in Biofilms

**Fig. 6:**
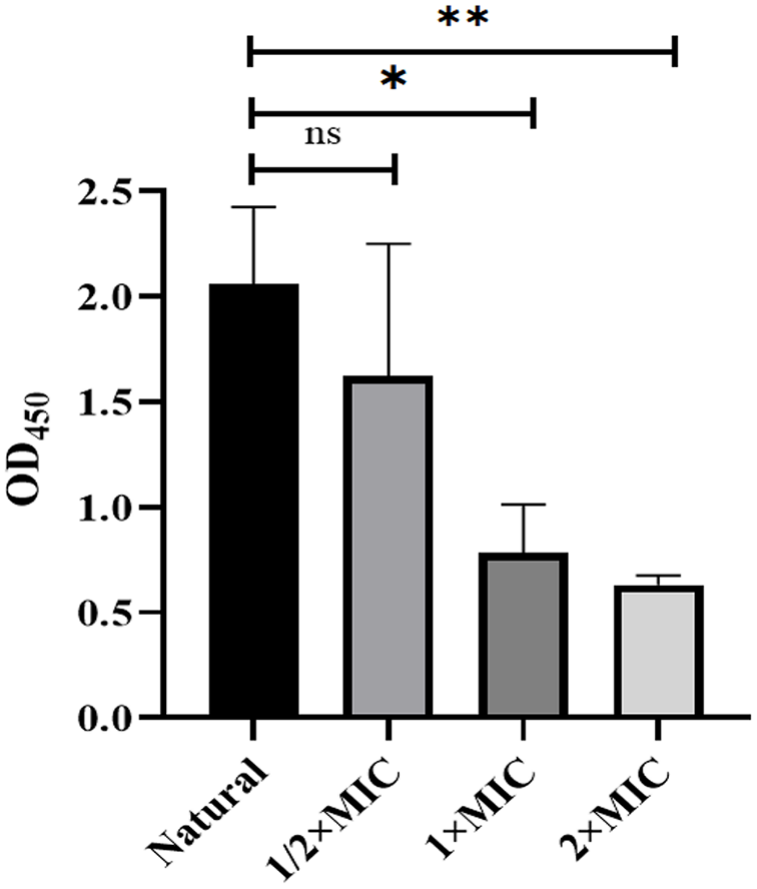
CTE reduction of bacterial viability within *S. aureus* biofilms. The OD_450_ values were measured by ultraviolet and visible spectrophotometer. Data are expressed as the mean values ± SD, and *n* = 3 in each group; *p < 0.05, **p < 0.01, ns: no statistical difference, vs. the natural.

The viability of *S. aureus* embedded in biofilms was tested by CCK8 assay. The results suggested that 1 × MIC and 2 × MIC of CTE had the capability to significantly kill the *S. aureus* embedded in biofilms (Fig. 6).

### Effect of CTE on the Cell Wall Integrity of *S. aureus*

**Fig. 7:**
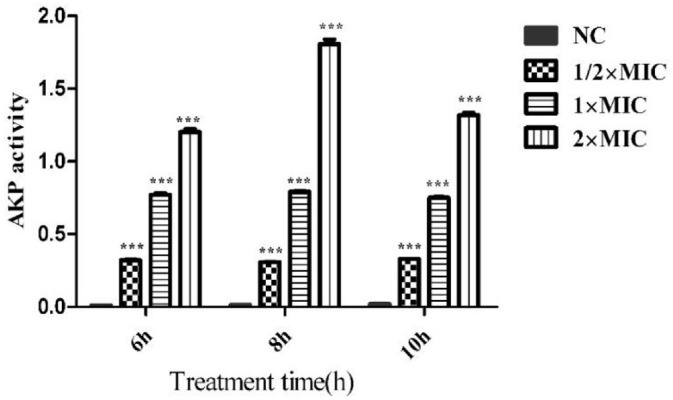
Extracellular AKP of *S. aureus* after CTE treatment. The optical densities at 405 nm (OD_405_) were determined by ultraviolet and visible spectrophotometer. Data are expressed as the mean values ± SD, and *n* = 3 in each group; ***p < 0.001, vs. the NC.

AKP exists between the cell membrane and the cell wall and is an important indicator for evaluating cell wall integrity. As shown in Fig. 7, the AKP activity in the culture supernatant of *S. aureus* was increased at the same time points as the treatment concentration increased. However, at the same treatment concentration, longer incubation times did not increase the AKP activity. The activity was highest at 2 × MIC at each time point, suggesting that a relatively high concentration of CTE could effectively destroy the cell wall of *S. aureus*.

### CTE Disrupts the Membrane Integrity of *S. aureus*

**Fig. 8:**
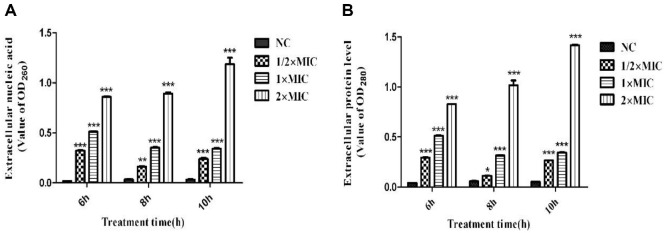
Extracellular nucleic acids and proteins of *S. aureus* after CTE treatment. The absorbance of the supernatants was determined (at wavelength of 260 nm or 280 nm) by ultraviolet and visible spectrophotometer. (**A**) Extracellular nucleic acids; (**B**) Extracellular proteins. Data are expressed as the mean values ± SD, and *n* = 3 in each group; *p < 0.05, **p < 0.01,***p < 0.001, vs. the NC.

Most proteins and nucleic acids are located inside of cells. Thus, proteins and nucleic acids that leak out of cells can serve as important indicators for evaluating the membrane integrity. As shown in Fig. 8, when *S. aureus* was cultured with 1/2 × MIC, 1 × MIC, and 2 × MIC of CTE for 6.0, 8.0, and 10.0 h, the proteins and nucleic acids that leaked out of the cells were increased, suggesting that CTE could effectively damage the membrane of *S. aureus*.

### CTE Increases the Intracellular ROS Content of *S. aureus*

**Fig. 9:**
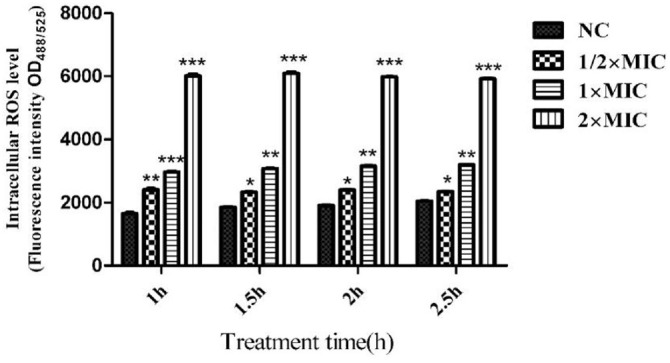
Intracellular ROS in *S. aureus* after CTE treatment. The fluorescence excitation/emission wavelengths were 488 nm/525 nm. Data are expressed as the mean values ± SD, and *n* = 3 in each group; *p < 0.05, **p < 0.01, ***p < 0.001, vs. the NC.

ROS are an important indicator that reflect the state of cellular activity. After *S. aureus* was treated with CTE for 1.0, 1.5, 2.0, and 2.5 h, the intracellular ROS were measured using a BioTek Synergy H1 microplate reader at fluorescence excitation/emission wavelengths of 488 nm/525 nm. At 1.0, 1.5, 2.0, and 2.5 h, the ROS contents in *S. aureus* cells were obviously increased compared with the control group after treatment with 1/2 × MIC, 1 × MIC, and 2 × MIC of CTE (Fig. 9). The results suggested that *S. aureus* treated by CTE produced a large amount of ROS, thereby demonstrating that CTE could induce bacterial oxidative damage.

### CTE Regulates Biofilm Formation-Related Gene Transcription

**Fig. 10:**
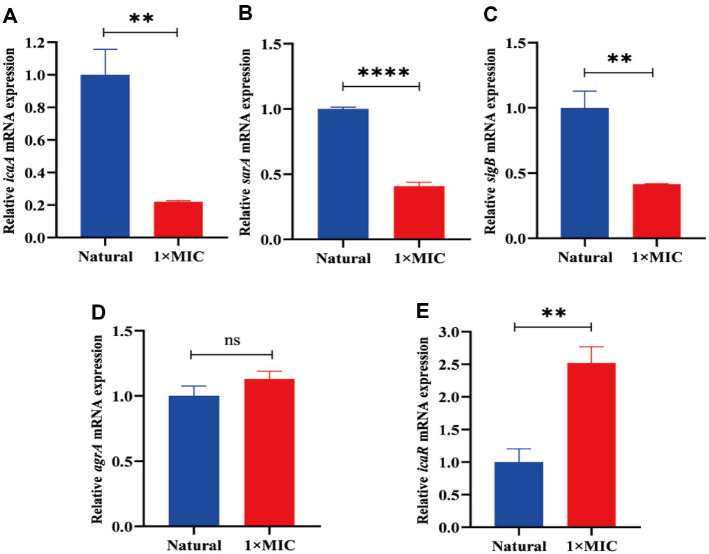
RT-qPCR analysis of mRNA levels of *icaA*, *sarA*, *sigB*, *agrA* and *icaR* after CTE treatment. *S. aureus* cells were treated with or without 1 × MIC of CTE for 12h. Total RNA was extracted, and then the expression of (**A**) *icaA*, (**B**) *sarA*, (**C**) *sigB*, (**D**) *agrA*, and (**E**) *icaR* was evaluated by RT-qPCR. Data are expressed as the mean values ± SD; **p < 0.01, ****p < 0.0001, ns: no statistical difference, vs. the natural.

IcaA, *sarA*, *sigB*, *agrA* and *icaR* genes play important roles in biofilm formation. To explore the role of CTE in regulating *icaA*, *sarA*, *sigB*, *agrA* and *icaR* gene transcription, *S. aureus* was cultured with 1 × MIC of CTE for 12 h, RNA was isolated, and the RT-qPCR was performed to assess gene transcription. The results indicated that the *icaA*, *sarA* and *sigB* mRNA levels were significantly downregulated, whereas the *icaR* mRNA level was obviously upregulated. Compared with the control group, the mRNA level of *agrA* in the CTE treated group was higher, but there was no statistical difference (Fig. 10). These data demonstrated that inhibition of *S. aureus* biofilm formation by CTE was achieved by regulating *icaA*, *sarA*, *sigB*, and *icaR* gene transcription.

## Discussion

Bovine mastitis is an inflammation of the mammary gland in diary cows [21], and *S. aureus* is an important pathogen causing this disease [20, 22]. In recent years, antibiotics have served as the main medicine for treating bovine mastitis [23]. However, owing to their overuse, *S. aureus* has developed resistance to many antibiotics, and the development of new treatments for bovine *S. aureus* mastitis is urgently needed.

Some plant extracts can induce cell microstructure damage, thereby playing an antibacterial role. In addition, plant extracts have the characteristics of low toxicity, no drug resistance and high safety, making them ideal alternatives for antibiotics [24]. *Clerodendron cyrtophyllum* Turcz. is a traditional Chinese medicine for treating various diseases, including human breast inflammation. Our previous study showed that CTE contains 18 different compounds, including luteoloside, acteoside, luteolin and stigmasterol [25]. Because luteolin and stigmasterol have antibacterial and anti-inflammatory activities, and luteoloside and acteoside have anti-inflammatory activities, we analyzed the effects of CTE on *S. aureus* and *S. aureus* mastitis. The results suggested that CTE has antibacterial activities against *S. aureus* from bovine mastitis and inhibited biofilm formation, providing evidence for the application of CTE in preventing and treating bovine mastitis.

The mechanisms of plant extracts against *S. aureus* from human and animal have been extensively studied, especially in terms of cellular permeabilities. Extracts damage the cell wall, causing cellular leakage and inducing bacterial cell death. Generally, AKP is located between the bacterial cell wall and cell membrane, and is difficult to detect outside the cell if the bacteria are intact [26]. Therefore, AKP is an important indicator for evaluating cell wall integrity. The effects of CTE on *S. aureus* cell wall were tested, and the results showed that CTE promoted the leakage of intracellular AKP, suggesting that the *S. aureus* cell wall was damaged. In addition to the cell wall, the cell membrane is another important protective barrier in bacteria [27]. Once the permeability of the cell membrane increases, intracellular substances will leak out, resulting in inhibited bacterial growth or apoptosis. In the present study, the effects of CTE on the membrane permeability of *S. aureus* were analyzed by examining extracellular nucleic acids and proteins. The results demonstrated that the extracellular nucleic acids and proteins content were significantly increased, indicating that CTE caused damage to the cell membrane of *S. aureus*. ROS are an important indicator for assaying the cell state [28]. When cells are subjected to adverse stimuli, the levels of ROS will rapidly increase. If the ROS content in *S. aureus* cells is increased, this in turn facilitates disruption of the bacterial cell membrane [29]. The findings of this study revealed that the intracellular ROS of *S. aureus* were greatly increased after treatment with CTE, suggesting that the inhibition of CTE on the growth of *S. aureus* might be due to ROS-mediated cell membrane disruption.

At present, most microbial infections are caused by biofilms. Biofilm is difficult to eradicate, because it can prevent antibiotics from entering, so that the bacteria inside the biofilm survive [30, 31]. Therefore, the formation of biofilm is an important factor that leads to drug-resistant bacteria emergence. Many of the complications caused by *S. aureus* have been reported to be chronic biofilm-like infections [32]. As mentioned above, the development of novel and effective antibiofilm agents has become necessary [33]. Inhibiting biofilm formation is crucial in finding new strategies to combat drug resistance [34]. In this study, the addition of CTE in *S. aureus* suspension inhibited the formation of biofilms and decreased the viability of *S. aureus* embedded in biofilms, indicating that CTE has potential as an antimicrobial for treating antibiotic-resistant bovine *S. aureus* mastitis.

To further explore the mechanisms of CTE inhibition of *S. aureus* biofilm formation, the expression of biofilm formation-related genes were determined by RT-qPCR. Alternative sigma factor B (SigB) plays essential roles in regulating biofilm formation. The upregulation of SigB is usually accompanied by increased biofilm formation, whereas *sigB* gene deletion mutant disables biofilm production in *S. aureus* [35, 36]. In this experiment, *S. aureus* was treated with CTE, and the mRNA level of *sigB* was downregulated. Staphylococcal accessory regulator A (SarA) is a main controller for biofilm formation, and *sarA* mutant displayed impaired biofilm formation [37]. The expression of *sarA* leads to enhanced biofilm formation by triggering the upregulation of cell wall-associated proteins [38]. After CTE treatment, the expression of *sarA* was decreased in *S. aureus*. The *icaA* gene is involved in the synthesis of the main polysaccharides in the biofilm matrix and *icaA* deletion decreases in formation of biofilm [39, 40]. The present study proved that CTE inhibited *sarA* expresion in *S. aureus*. It was reported that *icaA* is negatively regulated by *IcaR* [41]. Deletion of *icaR* results in increased *icaA* transcription and enhances biofilm synthesis [42]. In this study, the mRNA level of *icaR* was significantly upregulated in CTE-treated *S. aureus*. In summary, CTE treatment resulted in downregulation of *icaA*, *sarA*, and *sigB*, and upregulation of *icaR* in *S. aureus*, thereby inhibiting biofilm formation. In addition, *in vivo* study indicated that CTE inhibited the inflammatory cell infiltration and acini destruction in mammary gland tissues of mice with mastitis (Fig. S3), suggesting that CTE treatment alleviated inflammation in *S. aureus*-induced mastitis. In the present study, the effect of CTE on *S. aureus* mastitis was analyzed only by histological analysis, which was far from sufficient. In future studies, the inflammatory factors (TNFα, IL-2, IL-6), the number of S aureus in mammary gland, and the underlying mechanisms need to be detected, which may provide a theoretical basis for CTE application in the treatment of *S. aureus* mastitis.

## Supplemental Materials

Supplementary data for this paper are available on-line only at http://jmb.or.kr.



## Figures and Tables

**Table 1:** Specific primers used for RT-qPCR.

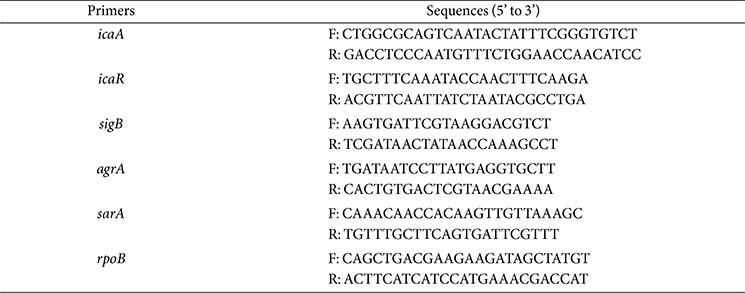

**Table 2:** Inhibitory zone diameter of CTE against *S. aureus*.

Treatment	Inhibitory zone diameter (mm)
DMSO	0 ± 0
Erythromycin (50 μg/ml)	24 ± 0.47
CTE (400 μg/ml)	13 ± 0.35
CTE (200 μg/ml)	7 ± 0.32
CTE (100 μg/ml, 50 μg/ml and 25 μg/ml)	0 ± 0

**Note:** An inhibitory zone diameter less than 7 mm indicated no inhibitory effect; *n* = 3 in each group. A diameter between 7 and 10 mm indicated low sensitivity; a diameter greater than 10 mm but less than 20 mm indicated moderate sensitivity; a diameter greater than 20 mm indicated high sensitivity.
