# Supplementary material for: Antibacterial and Antibiofilm Activity of Clerodendron Cyrtophyllum Turcz. Ethanolic Extracts against Staphylococcus aureus from Bovine Mastitis
Source: J Microbiol Biotechnol. 2025 Nov 27;35:e2506007. doi: 10.4014/jmb.2506.06007 (PMC12685583; doi:10.4014/jmb.2506.06007)
Supplement: Supplementary file 1 [file jmb-35-e2506007-supple.pdf]

J. Microbiol. Biotechnol. 2025. 35: e2506007

https://doi.org/10.4014/jmb.2506.06007

1. Materials and Methods

1.1. *S. aureus* Strain Identification

The strain was identified by PCR amplifications. The DNA was extracted using a plasmid DNA extraction kit, and a universal *S. aureus* 16*S rRNA* gene primer (Table.S1) was used to amplified the strain 16*S rRNA* gene. The PCR product was tested by 1% agarose gel and send to Shanghai Sangon Biotech Co., Ltd for sequencing.

Table. S1 PCR amplification primers

	Forward Primer（5’→3’）	Reverse Primer（5’→3’）
16S *rRNA* gene	ACTCCTACGGGAGGCAGCAG	GGACTACHVGGGTWTCTAAT

1.2. Animals and Mastitis Model

1.2.1. Animals

BALB/c mice (22~25 g, 6~7 weeks) were provided by Changsha Tianqin Biotechnology Co., Ltd. The mice were bred in a cage with one male and two females at 23 °C-25 °C with 12h light/dark cycle, and supplied with plenty of food and water. Female mice were transferred to separate cages when they pregnant. All animal procedures were approved by the Ethics Committee of Guizhou Medical University (No: 2400293).

1.2.2. Mouse Mastitis Model

A total of 16 lactating female BALB/c mice were anesthetized by pentobarbital (75 mg/kg). The fourth pair of mammary glands of the mice were disinfected with 75% alcohol and surgery cut off 1mm of the nipples, and then injected with 50uL *S. aureus* solution (2×10^5 CFU/mL). Subsequently, mice were randomly divided into 4 groups: normal saline treated group, positive control group (Dexamethasone, 5 mg/kg), low-dose CTE group (3.6 g/kg), high-dose CTE group (4.8 g/kg). Mice were given CTE or

normal saline by intragastric administration once a day for 6 days. On the 7th day, all mice were sacrificed and the mammary tissue were collected for histopathological detection.

1.2.3. Histological Evaluation of Mastitis

Mammary tissues were fixed with 4 % paraformaldehyde, embedded in paraffin and cut into 5μm-thick sections. And then the sections were stained with hematoxylin and eosin (HE). Histopathological changes of mammary gland tissues were observed by light microscope. The mammary gland injury score was according to previously described[1].

2. Results

2.1. *S. aureus* Strain Identification

As shown in Figure S1. the fragment of *S. aureus*16*S rRNA* gene was obtained and it was about 500bp. After that, sequencing of the amplification product and compared with the National Center for Biotechnology Information (NCBI) public database using the BLAST tool (http://www. ncbi. nlm. nih. gov /BLAST/). As shown in Figure S2, sequence of the amplification product obtained from *the strain* showed high similarity with the *S. aureus* 16*S rRNA* gene*,* these data indicated that the strain be tested was *S. aureus.*

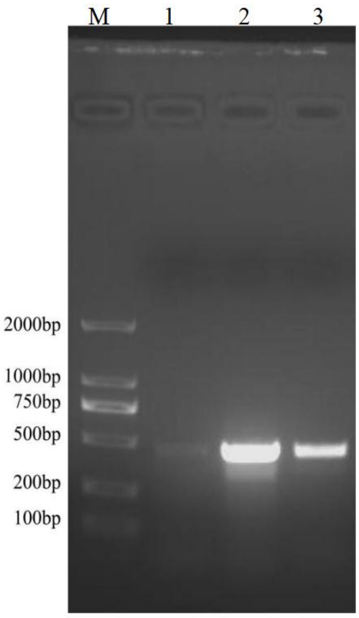

Fig. S1. The 16S rRNA gene of the tested strain amplified by PCR

Note: M: DL2000 DNA Marker; Lane 1-3: PCR amplification products.

Description	Scientific Name	Common Name	Taxid	Max Score	Total Score	Query Cover	E value	Per. Ident	Acc. Len	Accession
Staphylococcus aureus strain MRSA-3 16S ribosomal RNA gene, partial sequence	Staphyl...	NA	1280	1832	1832	99%	0.0	99.80%	1455	OP824647.1
Staphylococcus argenteus strain XM8 16S ribosomal RNA gene, partial sequence	Staphyl...	NA	985002	1831	1831	99%	0.0	99.90%	1454	MT023385.1
Staphylococcus aureus strain Cow 1 16S ribosomal RNA gene, partial sequence	Staphyl...	NA	1280	1831	1831	99%	0.0	99.90%	1064	ON138912.1
Staphylococcus aureus strain 4N112 16S ribosomal RNA gene, partial sequence	Staphyl...	NA	1280	1829	1829	99%	0.0	99.80%	1082	OM935820.1
Staphylococcus aureus strain O35 16S ribosomal RNA gene, partial sequence	Staphyl...	NA	1280	1827	1827	99%	0.0	99.70%	1255	OP776715.1
Staphylococcus aureus strain RM_AST_SA006 16S ribosomal RNA gene, partial sequence	Staphyl...	NA	1280	1825	1825	99%	0.0	99.80%	1448	MK809241.1
Staphylococcus aureus strain AM 16S ribosomal RNA gene, partial sequence	Staphyl...	NA	1280	1825	1825	99%	0.0	99.80%	1462	MG230264.1
Staphylococcus aureus strain HN-5 16S ribosomal RNA gene, partial sequence	Staphyl...	NA	1280	1825	1825	99%	0.0	99.80%	1462	KT003251.1
Staphylococcus aureus strain B0021-01F 16S ribosomal RNA gene, partial sequence	Staphyl...	NA	1280	1823	1823	99%	0.0	99.80%	1274	MH447002.1
Staphylococcus aureus strain RCB1010 16S ribosomal RNA gene, partial sequence	Staphyl...	NA	1280	1823	1823	99%	0.0	99.80%	1451	KT261222.1
Staphylococcus aureus strain CICC 10384 16S ribosomal RNA gene, partial sequence	Staphyl...	NA	1280	1823	1823	99%	0.0	99.80%	1479	KJ643929.1
Staphylococcus aureus strain MRSA-5043 16S ribosomal RNA gene, partial sequence	Staphyl...	NA	1280	1821	1821	99%	0.0	99.70%	1458	MT250912.1
Staphylococcus aureus strain DSM 20231 (Type) 16S ribosomal RNA gene, partial sequence	Staphyl...	NA	1280	1821	1821	99%	0.0	99.70%	1452	MN652637.1
Staphylococcus aureus strain FDAARGOS_40 16S ribosomal RNA gene, partial sequence	Staphyl...	NA	1280	1821	1821	99%	0.0	99.70%	1452	MN650260.1
Staphylococcus aureus strain MRSA-1 16S ribosomal RNA gene, partial sequence	Staphyl...	NA	1280	1821	1821	99%	0.0	99.80%	1454	OP824645.1
Staphylococcus aureus strain O43 16S ribosomal RNA gene, partial sequence	Staphyl...	NA	1280	1821	1821	99%	0.0	99.70%	1295	OP776719.1
Staphylococcus aureus strain SA-1 16S ribosomal RNA gene, partial sequence	Staphyl...	NA	1280	1821	1821	99%	0.0	99.70%	1458	OP604331.1
Staphylococcus aureus strain Cow 7 16S ribosomal RNA gene, partial sequence	Staphyl...	NA	1280	1821	1821	99%	0.0	99.70%	1043	ON138913.1

Fig. S2. The 16*S rRNA* gene of the tested strain compared with the *S. aureus* using the BLAST tool (http://www. ncbi. nlm. nih. gov /BLAST/).

2.2. CTE Alleviates *S. aureus* Induced Mastitis

The therapeutic effect of CTE on *S. aureus* induced mastitis was evaluated by HE staining. In the *S. aureus* group, some of the mammary acini were incomplete, and inflammatory cells increased in mammary tissues compared with CTE treated group and DEX treated group (Figure S3. A-D). However, CTE and DEX alleviated the pathological injury induced by *S. aureus*, the inflammatory cell infiltration and acini destruction were decreased (Figure S3. A-D). The histological scores revealed that CTE relieved *S. aureus* induced mastitis (Figure S3. E). The results demonstrated that CTE treatment alleviated *S. aureus* induced mastitis.

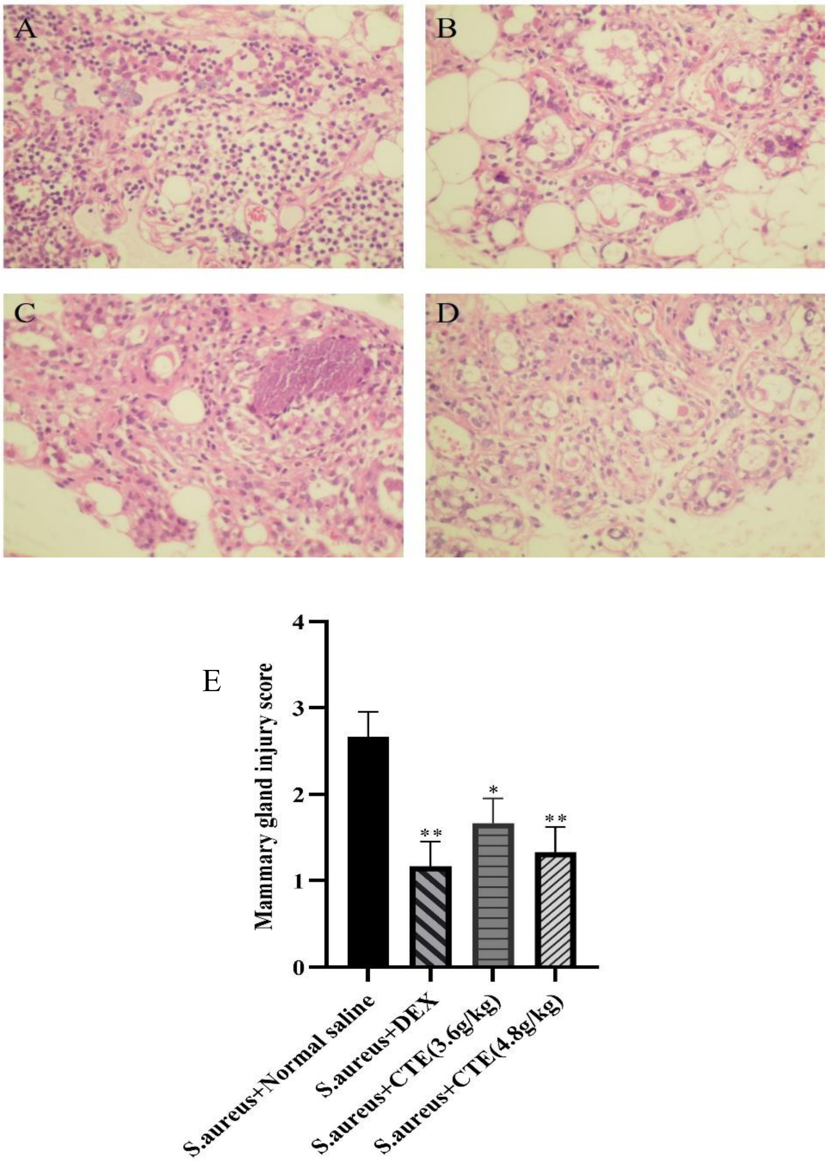

Fig. S3. Effects of CTE on *S. aureus* induced mastitis. Mamary gland tissue was stained by hematoxylin and eosin (HE staining, 5 × 40). (A) *S. aureus* + Normal saline; (B) *S. aureus* + DEX (5 mg/kg); (C) *S. aureus* + CTE (3.6g/kg); (D)*S. aureus* + CTE(4.8g/kg); (E) Histological scores. Data are expressed as the mean values ± SD, and n = 3 in each group. **P* < 0.05, ***P* < 0.01, vs the *S. aureus* + Normal saline.

参考文献

1. Guo W, Liu B, Hu G, Kan X, Li Y, Gong Q, *et al.* 2019. Vanillin protects the blood milk barrier and inhibits the inflammatory response in LPS-induced mastitis in mice. *Toxicology and Applied Pharmacology.* 365: 9-18.
